# Impacts of Partial Substitution of Chemical Fertilizer with Organic Fertilizer on Soil Organic Carbon Composition, Enzyme Activity, and Grain Yield in Wheat–Maize Rotation

**DOI:** 10.3390/life13091929

**Published:** 2023-09-18

**Authors:** Xiaoliang Li, Junchao Fang, Hiba Shagahaleh, Jianfei Wang, Amar Ali Adam Hamad, Yousef Alhaj Hamoud

**Affiliations:** 1 School of Resources and Environment, Anhui University of Science and Technology, Fengyang 233100, China; 2 Key Laboratory of Bioorganic Fertilizer Creation, Ministry of Agriculture, Bengbu 233400, China; 3 College of Environmental, Hohai University, Nanjing 210098, China; 4 College of Hydrology and Water Resources, Hohai University, Nanjing 210098, China

**Keywords:** inorganic fertilizers, soil organic carbon, enzyme activity, humus, replacement, crop rotation

## Abstract

This study explored the effect of the long-term partial replacement of chemical fertilizer with organic fertilizer on soil organic carbon composition, enzyme activity, and crop yields in the wheat–maize rotation area of northern Anhui, China. This study also specified the proper amount of organic fertilizer replacement that should be used for chemical fertilizer. Different fertilization modes were used (no fertilization, CK; chemical fertilizer, CF; chemical fertilizer and straw returning, CF + S; chemical fertilizer, straw returning, and straw decomposition agent, CF + S + DA; 70% chemical fertilizer and 50% organic fertilizer, 70% CF + 50% OF; 70% chemical fertilizer, 50% organic fertilizer and straw returning, 70% CF + 50% OF + S; 50% chemical fertilizer and 100% organic fertilizer, 50% CF + 100% OF; and 50% chemical fertilizer, 100% organic fertilizer, and straw returning, 50% CF + 100% OF + S). Variations in the organic carbon composition, enzyme activity, soil pH, and crop yields in the wheat–maize rotation under different fertilization treatments were analyzed. The results showed that the replacement of chemical fertilizer with organic fertilizer results in improved crop yields in wheat–maize rotation. The long-term partial replacement of chemical fertilizer with organic fertilizer can increase the quality of soil humus, alleviate soil acidification, and improve soil enzyme activity. Straw returning and organic fertilizer application can considerably raise the activities of urease, acid phosphatase, and nitrate reductase in soil. The soil pH of the CF treatment was reduced compared to the CK treatment, while organic fertilizer application alleviated soil acidification when compared to CF treatment. Organic fertilization increases the total organic carbon content of the soil, which was 19.6~85.5% higher than in the CK treatment. Applying straw and organic fertilizer significantly increased the ratio of the humic/fulvic acid in the soil. The soil active carbon forms of the soil with the application of organic fertilizer and straw returning were significantly higher than those of the CK and CF treatments. This study suggests that the optimal fertilizer management option in northern Anhui’s wheat–maize rotation area is to replace 50% of the chemical fertilizer with organic fertilizer, and to fully return straw to the field. This would include 150 kg N h·m^−2^, 60 kg P_2_O_5_ h·m^−2^, 50 kg K_2_O h·m^−2^, 6000 kg organic fertilizer h·m^−2^, and full straw return to the field.

## 1. Introduction

The Yangtze River Basin is China’s leading agriculture-producing zone, which accounts for 39.1% of the country’s total cultivated area [1]. High agricultural yields in this region are fundamental to warrant food safety in China. Therefore, the inputs of chemical fertilizers have increased significantly to increase crop yields [2,3]. However, a further increase in chemical fertilizer application is unlikely to be effective in continuously increasing crop yields [4]. Long-term chemical fertilizer application wastes resources, enlarges production costs, and increases negative environmental impacts [5,6]. The phosphorous fertilizer input efficiency of interest is useful as a metric. The phosphorous fertilizer input efficiency is low due to a great amount of phosphorous either being lost by runoff and leakage, or fixed by other soil minerals [2,7]. What is known about potassium fertilizer input efficiency is that if it is low, then a large amount of potassium is fixed or leaked [2]. Although the focus on chemical fertilizer input efficiency is on nitrogen, nitrogen input efficiency is very low as a large amount of nitrogen is lost to the environment [8]. The nitrogen emissions due to water pollution in China’s seven main river basins account for 89.5% of the country’s entire nitrogen emissions. Moreover, the nitrogen emissions from agricultural water bodies reached 141.49 Mt, where poultry, crop, and livestock farming activities accounted for more than 90% of the total emissions [9]. Therefore, chemical fertilizer input efficiency will continue to decrease, and the effect of chemical fertilization on cereal yields increase will regularly decline. Thus, the development of fertilizer-efficient technologies should be introduced to match high grain yields and for the large fertilizer utilization of cereals.

In China, livestock manure, poultry, and cake fertilizer represent 67% of organic fertilizer resources, which reaches nearly 50 Mt nitrogen, phosphorous, and potassium nutrients. Only 50% of the organic fertilizer resources are returned to fields [10]. However, the extreme input of organic fertilizer resources can cause serious environmental problems and a waste of valuable fertilizer resources. Previous studies have focused on the mechanism of soil fertilization and soil enzyme activity. For instance, Ying Xin et al. [5] clarified that deep tillage with an appropriate application of organic fertilizer or biochar can effectively improve the properties of lime concretion black soil. Also, Wang Wei et al. [4] found that applying exogenous organic carbon in the field can promote the distribution and accumulation of organic carbon in large-size aggregates, thus improving soil structure and increasing soil organic carbon contents. Furthermore, Cao Wei et al. [6] found that different organic fertilization treatments had a significant effect on the composition and structure of water-soluble organic matter in lime concretion black soil. Moreover, the combined application of straw return and organic fertilizer significantly increased the content of soil-soluble organic carbon and enhanced the stability of dissolved organic carbon content [6].

In China, the straw residue is greater than 900 Mt·year^−1^. However, only a slight quantity of stubble collected post-harvesting is employed in soil improvement and fiber production, while the main quantity of the straw is burned [11,12]. Wheat–maize rotation is common in China, and covering the soil surface using straw is a popular farming method. Returning straw residues to the soil decreases evaporation from the soil surface, improving soil productivity and crop yields [13,14]. However, cropping practices regarding pest control are harder to control in straw return farming. Instead, burying straw in soil improves the water storage of the topsoil [15]. Furthermore, burying straw in soil improves the soil structure and biological activities, as well as reduces soil degradation, regulates heat, and ultimately increases the productivity and sustainability of cropping systems [16]. However, there is very limited information on the effects of the partial replacement of chemical fertilizer with organic fertilizer, as well as on straw returning on enzyme activity and organic carbon contents in the black soil located in the northern Anhui region of China.

According to the National Bureau of Statistics, in 2021, the national wheat planting area was 23.6 Mt·h^−1^, and wheat yield accounted for 20.06% of the total grain yield [1]. Maize is the third most common crop after rice and wheat, and the Huang-Huai-Hai plains are one of China’s main agricultural producing areas. Therein, the lime concretion black soil is the dominant soil type, occupying an area of more than 4 million h·m^−2^ in China, and the northern Anhui region has the largest distribution area [17]. Therefore, this study hypothesizes that the partial replacement of chemical fertilizer with organic fertilizer and straw returning would improve the enzyme activity, total organic carbon content, and fertility of the black soil, thereby improving yields in the wheat–maize rotation in the northern Anhui region of China. This study is of great significance for improving the strategic policy of grain production and to maintain China’s grain security. This study also provides a theoretical basis and practical reference for reasonable fertilization in wheat–maize planting areas.

## 2. Materials and Methods

### 2.1. Characterization of the Research Area and Soil Properties

The long-term field experiment of this paper (2011–2022) was conducted at the Agricultural Science Research Institute of Linquan, Fuyang (33.06758° N 115.26232° E), Anhui, China. The experiment area belongs to the continental warm, temperate semi-humid monsoon climate zone. It has a mild climate, moderate rainfall, sufficient sunshine, and four distinct seasons. It is cold and rainy in spring, dry and a small degree of snow in winter, and hot and rainy in summer. The average lowest temperature is 12 °C, the average highest temperature is 22 °C, and the average annual temperature is 15 °C. The soil class in this site is lime concretion black soil, and the cultivation approach is wheat–maize rotation. The soil physiochemical properties before the experiment were as follows: clay 9.97%, silt 47.88%, sand 42.15%, the soil pH value was 5.72, the total organic carbon was 7.33 g·kg^−1^, the available nitrogen content was 80.16 g·kg^−1^, the available phosphorus content was 16.92 g·kg^−1^, the available potassium content was 116.70 g·kg^−1^, and the total phosphorus content was 0.36 g·kg^−1^.

### 2.2. Experimental Design, Cropping Practices, and Treatments

The experiment used a single-factor randomized block design with 8 treatments and 4 replicates. The plot area was 50 m^2^ (5 m × 10 m); in addition, a 0.5 m protection row and a 1 m sidewalk was set between the plots. The wheat variety named Fengdecun was selected as the test wheat, and the maize variety named Xianyu335 was selected for use as the test maize. The test wheat and maize were sown according to the recommendations of the selected varieties, as well as per the weather conditions of the experimental location in the specific year. The seedlings of wheat and maize were planted at rates of 20 plants/m^2^ and 5 plants/m^2^, respectively, with row intervals of 20 and 30 cm, as well as planting intervals of 20 and 30 cm.

The chemical fertilizers used in the experiment were urea (46% N), calcium superphosphate (16% P_2_O_5_), and potassium sulfate (50% K_2_O), which were all purchased from the market. Organic fertilizer (1.64% N, 3.60% P_2_O_5_, K_2_O, 45% organic matter) was provided by the Fuyang Yifeng Fertilizer Limited company, and Nanjing Agricultural University provided the straw decomposition agent. The amount of fertilizer used for each treatment is shown in Table 1.

The total N application rate in the wheat season was 300 kg·hm^−2^, of which 120 kg was applied to the soil as the base fertilizer, and 180 kg was applied to the soil as the topdressing fertilizer at the jointing stage and booting stage, respectively. In addition, the amounts of P_2_O_5_ and K_2_O were 120 and 100 kg·hm^−2^, respectively, and these were applied to the soil as the base fertilizer. A surface irrigation approach was performed for the two crops, water was provided sufficiently in the critical stage of effective growth stages, and this was then left to dry naturally before harvest. All disease, grass, pest control measures, and other field management measures were the same in all of the treatments, except for the fertilization treatments.

### 2.3. Data Collection

#### 2.3.1. Sample Collection

In June 2022, a five-point sampling method was used to collect 0–20 cm of topsoil in each experimental field plot. The soil samples were dried and mixed. Soil samples were passed through a 2 mm sieve to determine the soil EOC, DOC, and POC; the soil samples were also sieved through a 0.25 mm sieve to determine soil humic acid; and the soil samples were then sieved through a 0.149 mm sieve to determine the soil TOC. In August 2022, soil samples were collected using the same collection method. Fresh samples were stored in a refrigerator at 20 °C and used for soil enzyme activity determination within one week. The yield of wheat and maize was measured in June and October 2022, respectively.

#### 2.3.2. Determination of Soil Enzyme Activity

The soil urease activity was determined by the phenol sodium–sodium hypochlorite colorimetric method [9]. Soil phosphatase activity was determined by the phenyl disodium phosphate colorimetric method, and the degree of soil nitrate reductase was determined by the phenol sulfonic acid colorimetric method [9].

#### 2.3.3. Determination of Soil Organic Carbon Composition and pH

Soil pH was measured in 1:2.5 soil and deionized water extract while using a calibrated pH meter. The TOC content was quantified by the oxidation of potassium dichromate and by the titration of ferrous ammonium sulfate [18]. A 0.2 g soil sample was passed through a 0.25 mm sieve and placed in a test tube; then, 10.00 mL of 0.4 mol/L potassium dichromate-sulfuric acid solution was added. The tube was heated to 185 °C~190 °C for 30 min in the electric furnace. The digestion solution was transferred into a 250 mL flask. The tube was washed with water, and the washing solution was incorporated into the flask so that the total volume of the solution was controlled at 60 mL. Next, 3 drops of phenanthroline indicator were added, and the remaining potassium dichromate was titrated with an ammonium ferrous sulfate standard solution. The discoloration process of the solution was orange-blue-brown-red. The DOC was quantified in soil and water extract (1:2.5) at 25.8 °C after shaking for 60 min [19], as well as by centrifuging for 10 min at 4500 r·min^−1^ [20]. The resulting supernatant was filtered with a 0.45 mm tissue filter, and the resultant mixture was quantified by the oxidation of potassium dichromate and the titration of ferrous ammonium sulfate. The humic acid (HA), fulvic acid (FA), and humin (HM) contents were determined by the sodium pyrophosphate-sodium hydroxide extraction potassium dichromate oxidation capacity method. The POC was determined as described in an earlier study [21]. The EOC was measured by reacting finely ground air-dried soil samples with 333 mmol·L^−1^ of KMnO_4_ by shaking at 60 r·min^−1^ for 60 min [22]. The suspension was then centrifuged at 2000 r·min^−1^ for 5 min. The supernatant was diluted and measured by the spectrophotometer at 565 nm.

#### 2.3.4. Measurements of the Grain Yields

During the maturity period of the crops, a quantitative area of crops in each plot was manually harvested and threshed to measure each plot’s wheat and corn yields in each season. The dry wheat and corn grains had their moisture content measured. The yield of the wheat grains had a moisture content of 12.5%, and the corn grains had a moisture content of 14%. The grain yields were expressed in kg·hm^−2^.

### 2.4. Statistical Analysis

The data collected were processed with a single-factor variance in the SPSS22.0 for statistical analysis. A one-factor ANOVA model and the least significant difference (LSD) method were used to determine the significance of the difference between the treatments at the level of *p* ≤ 0.05. Pearson’s two-tailed test was used for correlation analysis, and Origin 2021 was used for mapping.

## 3. Results

### 3.1. The Effect of a Partial Replacement of Chemical Fertilizer with Organic Manure on Soil Enzyme Activity

**Figure 1:**
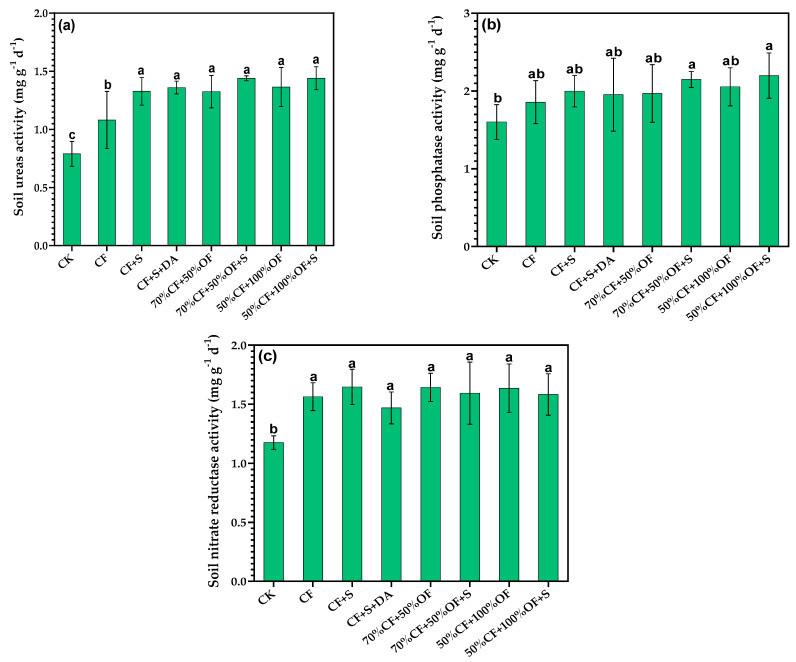
Effects of the partial fertilizer replacement with organic fertilizer and the return of straw on soil urease activity (**a**), soil phosphatase activity (**b**) and soil nitrate reductase activity (**c**). Different letters above bars indicate the significant differences between the treatments at the 0.05 level.

The experimental results showed significant differences in the urease activity that occurred in the different treatments (Figure 1a). Soil urease activity was the lowest in the CK treatment. Applying chemical fertilizer (CF) could significantly increase the urease activity of the soil, which was 36.9% higher than that of CK. The urease activity of soil with long-term exogenous carbon application (CF + S, CF + S + DA, 70% CF + 50% OF, 70% CF + 50% OF + S, 50% CF + 100% OF, and 50% CF + 100% OF + S) in the field was relatively high. There was no significant difference in the urease activity between the different exogenous carbon and the differing amounts of exogenous carbon.

The results showed significant differences in acid phosphatase activity among the different fertilization treatments (Figure 1b). A partial replacement of chemical fertilizer with organic fertilizer that is also combined with straw returning can significantly increase the acid phosphatase activity of soil. Among the tested treatments, 50% CF + 100% OF + S and 70% CF + 50% OF + S were the highest, respectively, followed by 50% CF + 100% OF, CF + S, 70% CF + 50% OF, CF + S + DA, and NPK treatments, while the CK treatment was the lowest.

The results of the field experiment (Figure 1c) showed that fertilization can significantly increase the nitrate reductase activity of soil. In the tested treatments, the nitrate reductase activity was the lowest in the CK treatment at only 1.176 mg g^−1^·d^−1^. The nitrate reductase activity of the soil treated with the CF, CF + S, CF + S + DA, 70% CF + 50% OF, 50% CF + 100% OF, 70% CF + 50% OF, and 50% CF + 100% OF + S treatments was significantly higher than that of the CK treatment, which increased by 32.9%, 40.0%, 24.9%, 39.7%, 39.0%, 35.5%, and 34.5%, respectively. There was no significant difference in nitrate reductase activity between the different fertilization treatments.

### 3.2. The Effect of a Partial Replacement of Chemical Fertilizer with Organic Manure on Soil pH and Soil Total Organic Carbon Content

**Figure 2:**
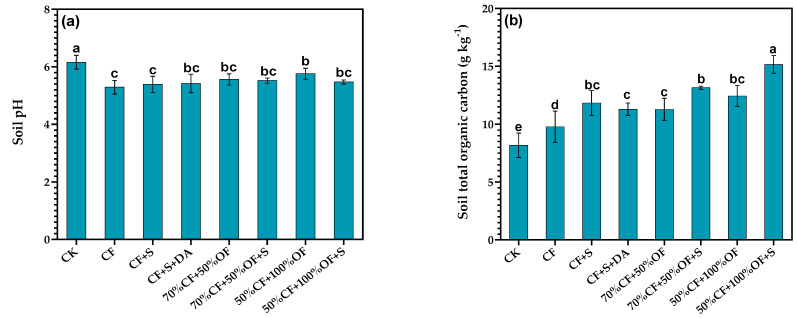
Effects of the partial replacement of chemical fertilizer by organic fertilizer and straw return on soil pH (**a**) and soil total organic carbon content (**b**). Different letters above bars indicate the significant differences between the treatments at the 0.05 level.

The results showed that the pH of the soil was significantly affected by the different fertilization treatments (Figure 2a). Before the test, the pH value of the soil was 5.72. After 11 years, the pH value of soil treated with CK was 6.16, which increased by 7.7%. The pH value of the soil in the NPK treatment was 5.29, which decreased by 7.45%, had an average annual decrease of 0.04, and the chemical fertilizer application to the soil had a significant acidification effect. Generally, straw returning and organic fertilizer application alone alleviated soil acidification.

**Figure 3:**
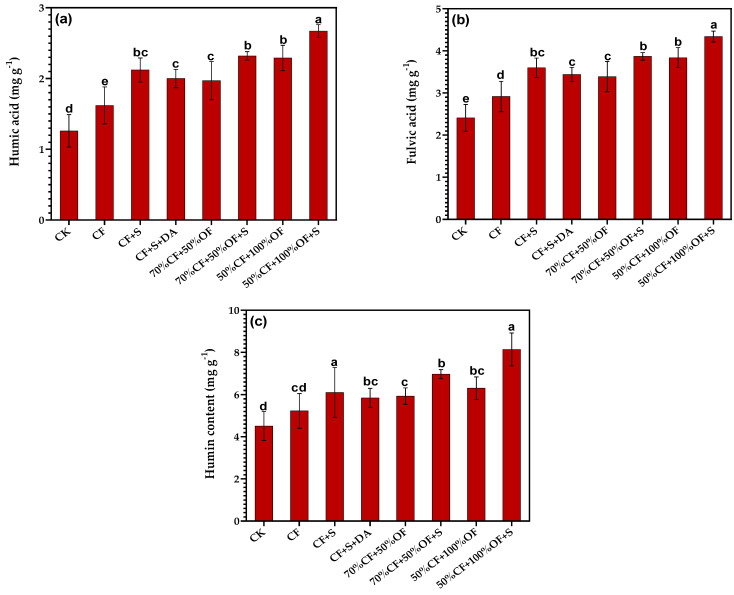
Effects of partial replacement of chemical fertilizer by organic fertilizer and straw return on the contents of soil humic acid (**a**), fulvic acid (**b**), and humin (**c**). Different letters above bars indicate significant differences among treatments at the 0.05 level.

The experiments showed that the TOC of lime concretion black soil was significantly affected by different fertilization treatments (Figure 3c). Before the experiment, the TOC of the lime concretion black soil was 7.33 g·kg^−1^. After 11 years, the TOC of soil treated with CK was 8.17 g·kg^−1^, thus an increase of 11.5%. The chemical fertilizer (CF) treatment, organic fertilizer substitution (70% CF + 50% of, 50% CF + 100% OF), and organic fertilizer substitution and straw returning (70% CF + 50% of + S, 50% CF + 100% OF + S) showed higher TOC values than those before the experiment and CK treatment. Therefore, the partial replacement of chemical fertilizer with organic fertilizer when combined with straw returning can significantly increase the TOC of lime concretion black soil, as well as significantly improve soil fertility.

### 3.3. The Effect of a Partial Replacement of Chemical Fertilizer with Organic Manure on Humus Carbon Fractions in Soil

The ANOVA analysis showed that the humic acid (HA) content in the 50% CF + 100% OF + S treatment was 2.67 g·kg^−1^, which was significantly higher than in other treatments (Figure 3a). It can be seen in Figure 3b that the fulvic acid (FA) contents in the 50% CF + 100% OF + S, 70% CF + 50% OF + S, 50% CF + 100% OF, CF + S, CF + S + DA, and 70% CF + 50% OF treatments were significantly higher than those of the NPK and CK treatments. It can be seen in Figure 3c that the humin (HM) content in the 50% CF + 100% OF + S treatment was 8.14 g·kg^−1^, which was significantly higher than that in other treatments.

Fertilization increases the HA and FA in soil TOC but will reduce the amount of HM (Table 2). Among the treatments, the HA/TOC was the highest in the 50% CF + 100% OF treatment at 18.42%. The proportion of HA in the soil treated with straw returning (CF + S, CF + S + DA), organic fertilizer replacement (70% CF + 50% OF, 50% CF + 100% OF), and organic fertilizer replacement + straw returning (70% CF + 50% OF + S, 50% CF + 100% OF + S) was significantly higher than that of the CK treatment. There was no significant difference in the HA/TOC between different treatments. The FA/TOC was the highest in the 50% CF + 100% OF treatment at 30.88%, and the lowest in the CK treatment at 29.52%. There was no significant difference in the FA/TOC between different treatments. The HM/TOC was the highest in the CK treatment at 55.17%, and the lowest in the 50% CF + 100% OF treatment at 50.70%. There was no significant difference in the HM/TOC between different treatments.

Significant differences in the HA/FA were found between the different treatments (Table 2). Among the test treatments, the 50% CF + 100% OF + S treatment had the highest increase at 61.46%. The proportion of HA in the soil treated with chemical fertilizer, straw returning (CF + S, CF + S + DA), organic fertilizer replacement (70% CF + 50% OF, 50% CF + 100% OF), and organic fertilizer replacement + straw returning (70% CF + 50% OF + S, 50% CF + 100% OF + S) was significantly higher than that of the CK treatment.

### 3.4. The Effect of a Partial Replacement of Chemical Fertilizer with Organic Manure on Active Organic Carbon Fractions in Soil

**Figure 4:**
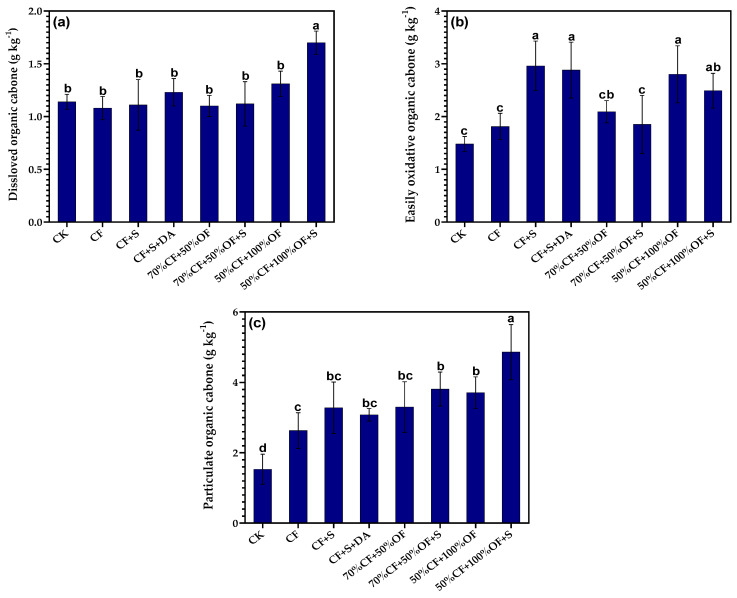
Effects of a partial replacement of chemical fertilizer with organic fertilizer when combined with straw return on the soil contents of dissolved organic carbon (**a**), easily oxidative carbon (**b**), and particulate organic carbon (**c**). Different letters above bars indicate significant differences between the treatments at the 0.05 level.

The DOC content in the 50% CF + 100% OF + S treatment was 1.70 g·kg^−1^, which was significantly higher than that found in other treatments. Having said this, there was no significant difference between the other treatments in this regard (Figure 4a). The EOC content of the CF + S treatment was the highest, reaching 2.96 g kg^−1^ (Figure 4b). The POC content of 50% CF + 100% OF + S treatment was significantly higher than that of other treatments with a content of 4.86 g·kg^−1^. The treatment of applying exogenous carbon was higher than the NPK and CK treatments in terms of POC content (Figure 4c).

In the straw returning treatment, the DOC of the CF + S treatment was 2.6% lower than that of the CK treatment. In addition, the CF + S + DA treatment was 7.9% higher than that of the CK treatment. The EOC of the CF + S and CF + S + DA treatment increased by 100.0% and 94.6%, respectively, when compared with the CK treatment. Moreover, the POC also increased by 114.4% and 101.3%, respectively, when compared with the CK treatment. This indicates that straw returning has little effect on the DOC in soil, but significantly affects the EOC and POC. In the organic fertilizer replacement treatment, the DOC of the 70% CF + 50% OF treatment was 3.5% lower than that of the CK treatment, and the EOC and POC were 41.2% and 89.2% higher, respectively, than those of the CK treatment. The DOC, EOC, and POC of the 50% CF + 100% OF treatment were 19.4%, 33.7%, and 12.6% higher than those of the 70% CF + 50% OF treatment, as well as 14.9%, 89.5%, and 143.0% higher than those of the CK treatment, respectively. In the treatment of organic fertilizer replacement + straw returning, the DOC of the 70% CF + 50% OF + S treatment was 1.8% and 1.8% higher than that of the 70% CF + 50% OF and CK treatments, respectively. In addition, that of the 50% CF + 100% OF + S treatment was 29.8% and 51.8% higher than that of the 50% CF + 100% OF and 70% CF + 50% OF + S treatments, respectively. The EOC of the 70% CF + 50% OF + S treatment increased by 11.5% and 25.0% when compared with the 70% CF + 50% OF and CK treatments, respectively. Furthermore, the 50% CF + 100% OF + S treatment increased by 11.1% and 34.6% when compared with the 50% CF + 100% OF and 70% CF + 50% OF + S treatments, respectively. The POC of the 70% CF + 50% OF + S treatment was 15.5% and 149.0% higher than that of the 70% CF + 50% OF and CK treatments, respectively. In addition, the 50% CF + 100% OF + S treatment was 31.0% and 27.6% higher than that of the 50% CF + 100% OF and 70% CF + 50% OF + S treatments, respectively. This shows that organic fertilizer replacement and organic fertilizer replacement + straw returning can significantly increase the content of EOC and POC in soil. A low replacement ratio of organic fertilizer and organic fertilizer + straw returning will reduce the DOC of soil, while a high replacement ratio of organic fertilizer and organic fertilizer + straw returning can increase the DOC of soil, as well as effectively increase the content of the EOC and POC in soil.

### 3.5. The Effect of a Partial Replacement of Chemical Fertilizer with Organic Manure on Yields of Maize and Wheat

**Figure 5:**
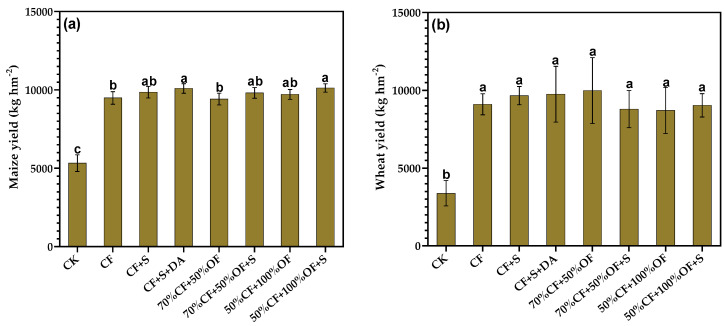
Effects of the partial replacement of chemical fertilizer by organic fertilizer and straw return on maize (**a**) and wheat (**b**) yields. Different letters above bars indicate the significant differences between the treatments at the 0.05 level.

The results showed that fertilization can significantly increase the yields of maize (Figure 5a) and wheat (Figure 5b). Among the treatments, the yields of maize and wheat were the lowest in the CK treatment. The yields of maize and wheat treated with NPK, CF + S, CF + S + DA, 70% CF + 50% OF, 50% CF + 100% OF, 70% CF + 50% OF, and 50% CF + 100% OF + S were significantly higher than that of the CK and chemical fertilizer treatments.

### 3.6. Correlation Analysis of Organic Carbon Components and Enzyme Activity in the Soil

Soil organic carbon and its components are one of the main factors affecting soil enzyme activity (Table 3). Soil urease activity was significantly positively correlated with the total organic carbon; three types of humus; and the POC, EOC, and HA/FA, as well as significantly correlated with HA/TOC. Soil acid phosphatase activity was positively correlated with the total organic carbon, HA, FA, POC, HA/FA, and HA/TOC. Soil nitrate reductase was significantly positively correlated with the HA, FA, HA/FA, and HA/TOC, as well as significantly positively correlated with the total organic carbon and POC.

There was a significant or extremely significant negative correlation between the soil organic carbon and its components and enzyme activity, which may be related to the change in the soil pH caused by fertilization (Table 3).

A significant or extremely significant positive correlation existed between the soil organic carbon and organic carbon fractions. There was a significant positive correlation between the TOC, HA, FA, and HM in the lime concretion black soil, a significant positive correlation between the DOC and POC, and a significant positive correlation between the POC and EOC (Table 3).

## 4. Discussion

### 4.1. Soil Enzyme Activity

After 11 years of testing, the partial substitution of chemical fertilizer with organic fertilizer boosted soil enzyme activity and enhanced the total organic carbon content of the soil, thereby increasing the grain yields in wheat–maize rotation. Soil enzyme activity is an important index through which to evaluate soil fertility and ecological environment quality [23]. The results showed that fertilization could significantly increase the activity of urease, phosphatase, and nitrate reductase in the soil. In particular, the activity of urease and phosphatase in the soil increased with the application of exogenous organic carbon. These results are attributed to the fact that the integration of straw and organic fertilizers can be rapidly exploited by soil microorganisms that are highly motivated. Equally, the activity of the soil enzymes was significantly or extremely significantly positively correlated with the TOC, HA, FA, and POC, which is consistent with previous studies’ results [24,25]. Moreover, compared with chemical fertilizers, straw and organic fertilizers can be rapidly utilized by plants and microorganisms, whereas the active organic carbon accounts for only a small part; most of the organic carbon nutrient release is a long-term process [26,27]. Therefore, in this study, the application of straw and organic fertilizers can increase soil organic carbon content and increase soil enzyme activity. Meanwhile, the increase in soil enzyme activity can promote the decomposition of soil organic carbon, increase the content of soil active organic carbon, increase the mineralized carbon in the soil, and provide sufficient nutrients for plant growth and development. The long-term application of chemical fertilizer could increase soil fertility. In this study, the long-term effect of straw and organic fertilizer application was greater than that in the NPK treatment as the exogenous organic carbon could enlarge the soil’s capability in conserving nutrients and make them available to the plant. The increase in the straw and organic carbon decomposition helped to promote ion exchange/absorption and thus increase soil fertility. Furthermore, straw is easy to degrade, thereby causing an increase in the EOC, TOC, and DOC contents in the soil. Thus, the role of straw and organic carbon degradation in modifying the dynamics of nutrients in the soil can be used to optimize crop production. Equally, several studies have reported that straw and organic carbon application improves organic matter and nutrient storage in soil [28].

### 4.2. Soil Acidification

Applying chemical fertilizers led to soil acidification, and the pH of the NPK treatment was lower than that of the CK treatment. In this experiment, the soil acidification of all treatments was enlarged compared with the original soil acidity, but the organic substitution treatment led to a slighter rise. These results were ascribed to the variations in the agrohydrological conditions, weather changes, and experimental years. Similarly, the study of Guo et al. [29] showed that the soil pH of farmland in China decreased considerably during 20 years of experimentation. In modern agriculture, with its high input and output, soil acidification has become the most common problem for cultivated land in the world [30]. However, in this study, soil acidification tended to be alleviated after applying straw and organic fertilizer. Applying organic fertilizer increased the soil pH on average compared with the CF treatment, and the effect of alleviating acidification was more significant with the increase in organic fertilizer, which is consistent with the research results of Zhang et al. [31]. Also, applying straw and organic fertilizer could increase soil organic matter and alleviate soil acidification [32,33].

### 4.3. Total Organic Carbon and Humus Carbon Fractions in Soil

Soil TOC is a key index through which to measure soil quality [16]. The results showed that fertilization could significantly increase the TOC of soil, and the effect of the exogenous organic carbon, such as straw and organic fertilizer, was more significant than that of the NPK treatment. With the increase in the proportion of organic fertilizer instead of chemical fertilizer, the content of soil TOC also increased. These results are because the long-term application of organic matter to soil increases the straw decomposition level and thus the TOC. Moreover, straw is easy to degrade, and it increasing the TOC content is consistent with the research results of an earlier study [34]. In this study, adding exogenous organic materials directly increased the soil carbon input, and the amount of newly formed organic carbon was greater than that of the soil organic carbon decomposition. Therefore, the TOC content of the soil increased. Thus, the partial replacement of chemical fertilizer with organic fertilizer and straw return can be an important option through which to improve soil quality, productivity, fertility, and crop production.

The soil HA/FA ratio indicates the degree of soil humus aggregation. A higher HA/FA ratio specifies a higher degree in the aggregation of humus [35]. Accordingly, our results prove that exogenous organic carbon can significantly increase a soil’s HA/FA ratio. Also, with the increase in the organic fertilizer application rate, the HA/FA ratio increased. Moreover, the HA fraction was less than that of the FA fraction, indicating that applying organic fertilizer and straw return could improve the degree of soil humification. Consistently, recent studies have shown that increasing the degree of soil humification could promote the condensation of FA into HA with a high degree of aromatization and complex structure. Therefore, the degree of polymerization and humification of soil humus increases significantly, thus improving the quality of the soil humus [36].

The soil DOC, EOC, and POC were the main indicators of soil active organic carbon [37]. They are active chemical components in the soil and are of great significance to the ecological environment. The results of this study showed that exogenous organic carbon affected the content of soil active organic carbon, where the contents of the DOC, EOC, and POC in the soil increased with the increase in straw and organic fertilizer application. These results can be attributed to the fact that the application of straw and organic fertilizers reduced the soil pH while enlarging the DOC, EOC, and POC contents of the soil due to the increased straw degeneration rates. Moreover, straw easily decays, thus enhancing the DOC, EOC, and POC contents. Consistently, microorganisms can rapidly utilize the soil’s active organic carbon fractions to improve soil microbial activity [38], thereby accelerating the decomposition and release of organic matter and the transformation and utilization of soil carbon. Also, organic fertilizer input provides a sufficient carbon source and energy for microorganisms, as well as directly promotes the growth and reproduction of microorganisms, improves microbial activity, and decomposes and releases more active organic carbon to increase soil DOC, EOC and POC content. Moreover, when the organic fertilizer enters the soil, it is combined with some sand particles, which promotes the decomposition of exogenous organic materials, the turnover of original organic materials, as well as the formation and transformation of POC.

### 4.4. Grain Production of Maize and Wheat

The development of maize and wheat under straw and organic fertilizer application was greater than those under the NPK and CK treatments. In this study, the partial substitution of chemical fertilizer with organic fertilizers can provide crop growth requirements, thereby increasing the number of grains per panicle and thus achieving higher maize and wheat yields. Equally, higher panicles and rice yield were achieved in enhancing the nutrient availability than in cases of reduced availability [39]. Moreover, aboveground total dry matter is a straight reflection of crop development. Therefore, the straw and organic fertilizer application could offer encouraging conditions to the plant, thereby supporting the growth of tillers and panicles, and thus biomass production, when compared to the NPK and CK treatments. Consistently, the modifications in the nutrient contents of the soil affected the nutrient supply of the plants, thus further impacting crop growth and yields [39,40]. After 11 test years, organic fertilizer increased the soil environment, stimulated the captivation of nutrients by wheat and maize, and thus increased the total biomass. Therefore, the wheat and maize yield enhancement was closely linked to nutrient uptake ability [41]. Correspondingly, favorable nutrient conditions for wheat were created under straw and organic carbon application conditions, where the yields were reliant on the plant growth traits, and the maize yield increased under organic carbon application conditions when compared to the control treatment [40]. This study proposes that optimal vegetative growth under straw and exogenous organic carbon application conditions plays a fundamental role in the high grain production of maize and wheat.

## 5. Conclusions

After 11 years of testing, substituting chemical fertilizer with organic fertilizer improved soil enzyme activity and increased the total organic carbon content of the soil. Specifically, the integration of chemical fertilizer, organic fertilizer, and straw returning alleviated soil acidification. This integration also increased the HA/FA, thereby enhancing the stability of total organic carbon in the soil; regulating the soil DOC, EOC, and POC; and increasing the activity of the soil microorganisms. Therefore, it can accelerate the transformation of the organic carbon in soil, as well as improve its fertility and crop yield. This study suggests that the integration of chemical fertilizer, organic fertilizer, and straw returning can be implemented to improve soil fertility, increase crop yield, and ensure sustainable soil utilization in the wheat–maize rotation areas in the northern Anhui province. Furthermore, this study also supports organic fertilizer as a long-term partial replacement for chemical fertilizer.

## Figures and Tables

**Table 1:** The amount of chemical fertilizer replaced with organic fertilizer used for each wheat and maize crop treatment.

Abbrev.	Treatment	Type and Dosage of Fertilizer (kg·hm^−2^)					
		N	P_2_O_5_	K_2_O	Straw Return	Organic Fertilizer	Straw Decay Agent
CK	Check	0	0	0	0	0	0
CF	Chemical fertilizer	652.17	750	200			0
CF + S	Chemical fertilizer + straw returning	652.17	750	200	Full dose	0	0
CF + S + DA	Chemical fertilizer + straw returning + straw decay agent	652.17	750	200	Full dose	0	150
70% CF + 50% OF	70% chemical fertilizer + 200 organic fertilizer	456.52	525	140	0	3000	0
70% CF + 50% OF + S	70% chemical fertilizer + 200 organic fertilizer + straw returning	456.52	525	140	Full dose	3000	0
50% CF + 100% OF	50% chemical fertilizer + 400 organic fertilizer	326.09	375	100	0	6000	0
50% CF + 100% OF + S	50% chemical fertilizer + 400 organic fertilizer + straw returning	326.09	375	100	Full dose	6000	0

**Table 2:** The ratio of humus carbon components for the different fertilization treatments.

Treatment	Humic/Fulvic Acid (HA/FA, %)	Humic Acid/TOC (HA/TOC, %)	Fulvic Acid/TOC (FA/TOC, %)	Humin/TOC (HM/TOC, %)
CK	51.85% ± 0.03 d	15.32% ± 0.02 b	29.52% ± 0.02 a	55.17% ± 0.04 a
CF	55.47% ± 0.02 c	16.60% ± 0.01 ab	29.92% ± 0.02 a	53.48% ± 0.03 a
CF + S	58.80% ± 0.01 b	18.05% ± 0.02 a	30.66% ± 0.04 a	51.29% ± 0.06 a
CF + S + DA	58.17% ± 0.01 b	17.74% ± 0.01 a	30.48% ± 0.01 a	51.79% ± 0.02 a
70% CF + 50% OF	57.98% ± 0.02 b	17.39% ± 0.01 a	29.97% ± 0.01 a	52.64% ± 0.02 a
70% CF + 50% OF + S	59.85% ± 0.01 ab	17.62% ± 0.01 a	29.44% ± 0.01 a	52.95% ± 0.01 a
50% CF + 100% OF	59.65% ± 0.01 ab	18.42% ± 0.00 a	30.88% ± 0.01 a	50.70% ± 0.01 a
50% CF + 100% OF + S	61.45% ± 0.01 a	17.65% ± 0.01 a	28.71% ± 0.02 a	53.64% ± 0.03 a

Note: Data are the mean value ± the standard deviation. The data followed by different letters after them indicate a significant difference between treatments at the 0.05 level.

**Table 3:** Correlations of the partial replacement of chemical fertilizer with organic fertilizer on soil enzyme activity, pH, and organic carbon components.

Soil Indicator	Urease Activity	Phosphatase Activity	NR Activity	pH	TOC	HA	FA	HM	DOC	POC	EOC	HA/FA	HA/TOC	FA/TOC	HM/TOC
Urease	1														
Phosphatase	0.411 *	1													
NR	0.445 *	0.335	1												
pH	−0.534 **	−0.356 *	−0.381 *	1											
TOC	0.841 **	0.455 **	0.410 *	−0.423 *	1										
HA	0.823 **	0.553 **	0.490 **	−0.440 *	0.949 **	1									
FA	0.825 **	0.556 **	0.492 **	−0.440 *	0.948 **	1.000 **	1								
HM	0.783 **	0.329	0.303	−0.372 *	0.960 **	0.822 **	0.820 **	1							
DOC	0.207	0.330	0.020	0.042	0.520 **	0.552 **	0.548 **	0.451 **	1						
POC	0.786 **	0.451 **	0.364 *	−0.459 **	0.946 **	0.894 **	0.893 **	0.911 **	0.513 **	1					
EOC	0.516 **	0.178	0.300	−0.417 *	0.496 **	0.546 **	0.549 **	0.408 *	0.232	0.501 **	1				
HA/FA	0.854 **	0.576 **	0.520 **	−0.526 **	0.913 **	0.976 **	0.976 **	0.779 **	0.452 **	0.866 **	0.555 **	1			
HA/TOC	0.438 *	0.542 **	0.449 **	−0.348	0.398 *	0.662 **	0.665 **	0.130	0.332	0.381 *	0.436 *	0.707 **	1		
FA/TOC	−0.147	0.246	0.161	−0.0219	−0.251	0.063	0.066	−0.507 **	0.068	−0.229	0.111	0.103	0.776 **	1	
HM/TOC	−0.122	0-.401 *	−0.307	0.178	−0.043	−0.352 *	−0.355 *	0.235	−0.198	−0.047	−0.272	−0.396 *	−0.929 **	−0.954 **	1

Note: NR, nitrate reductase; TOC, total organic carbon; HA, humic acid; FA, fulvic acid; HM, humin; DOC, dissolved organic carbon; POC, particulate organic carbon; and EOC, easily oxidative organic carbon. *, and ** represent the significant differences between the treatments at *p* ≤ 0.01, 0.05, and 0.001, respectively.

## Data Availability

The data presented in this study are available on request from the corresponding authors.
